# Resilient Phenotype in Chronic Mild Stress Paradigm Is Associated with Altered Expression Levels of miR-18a-5p and Serotonin 5-HT_1a_ Receptor in Dorsal Part of the Hippocampus

**DOI:** 10.1007/s12035-019-1622-2

**Published:** 2019-05-16

**Authors:** Dariusz Zurawek, Piotr Gruca, Lucyna Antkiewicz-Michaluk, Marta Dziedzicka-Wasylewska

**Affiliations:** 1 Faculty of Biochemistry, Biophysics and Biotechnology, Department of General Biochemistry, Jagiellonian University, Gronostajowa 7, 30-387 Krakow, Poland; 2 Polish Academy of Sciences, Department of Pharmacology, Institute of Pharmacology, 12 Smetna Street, 31-343 Krakow, Poland; 3 Polish Academy of Sciences, Department of Neurochemistry, Institute of Pharmacology, 12 Smetna Street, 31-343 Krakow, Poland

**Keywords:** miR-18a-5p, Stress, Resilient, Depression, Serotonin 5-HT1a receptor, Hippocampus

## Abstract

Disturbed serotonergic signaling in the hippocampus observed in many individuals vulnerable to stress has been suggested as one of the primary factors contributing to the development of depression. However, little is known about the physiology of the brain in the resilient phenotype. Resilient subjects maintain a positive mood and psychological balance despite being under the stress influence. In our study, we generated stress-vulnerable and resilient rats by using a chronic mild stress (CMS) paradigm. Using different molecular approaches, we revealed that resilient animals exhibited a significantly decreased expression level of miR-18a-5p and, in the same time, an elevated level of 5-HT1AR in dorsal, but not ventral, part of the hippocampus. Described biochemical changes were not observed in animals behaviorally vulnerable to stress. Further, in vitro analysis showed that miR-18a-5p may be a negative epigenetic regulator of 5-HT1AR since the treatment of adult hippocampal neurons with miR-18a-5p mimic significantly lowered the expression level of mRNA encoding 5-HT1AR. Moreover, bioinformatic analysis of potential target genes expressed in the hippocampus and being regulated by miR-18a-5p showed that this microRNA may regulate biological processes, such as axonogenesis, which are important in the functioning of the hippocampus in both rats and humans. All these molecular features may contribute to serotonergic homeostatic balance at the level of serotonin turnover observed in hippocampi of resilient but not stress-vulnerable rats. Delineation of further molecular and biochemical markers underlying resilience to stress may contribute to the development of new antidepressant strategies which will restore resilient phenotype in depressed patients.

**Electronic supplementary material:**

The online version of this article (10.1007/s12035-019-1622-2) contains supplementary material, which is available to authorized users.

## Introduction

Stress, defined as the biological response of an organism to life-threatening events, is an integral component of life. In the brain, short-term stress evokes a series of molecular changes responsible for coping with an unsafe situation. However, when the stressful events persist over a prolonged period of time, they lead to an allostatic load in the brain and, in effect, the loss of its homeostatic balance, which may result in the development of stress-related mood disorders such as depression. Nevertheless, in a human population, we can observe individuals who are much less prone to the negative influence of stress than others. These subjects are defined as resilient. Although resilient behavior has been extensively investigated in the context of psychological studies since the 1970s [1, 2], research aimed at understanding the molecular bases of this behavioral phenotype is sparse. Therefore, the expansion of neurobiological and molecular studies to investigate the resilience phenomenon seems to be of prime importance and may result in the development of more efficient antidepressant strategies based on the restoration of the resilient phenotype among depressed subjects.

Depressive disorders may be precipitated in an individual by an interaction between external stressful conditions and genetic and epigenetic predisposing factors [3–6]. In this context, much attention has been paid to microRNAs (miRNAs), which belong to a newly discovered class of pleiotropic, epigenetic regulators of a diverse set of biological processes [7]. Mature miRNAs are short (18–23 nucleotides), noncoding, single-stranded RNA transcripts that, among mammals, are highly evolutionarily conserved. miRNAs can affect gene expression patterns via degradation or inhibition of translation of their specific targets based on complementarity between the miRNA seed region and the targeted mRNA [8]. Recent evidence has shown impaired expression levels of different mature miRNAs in the brain of depressed suicides [9] as well as stressed animals [10, 11]. It has been observed that a maladapted stress response is associated with changes in the expression of many different miRNAs in the hippocampus [12, 13] and frontal cortex [14]. Moreover, elevated serum levels of miR-132 and miR-182 negatively correlated with serum levels of brain-derived neurotrophic factor (BDNF) in depressed patients [15]. Twelve weeks of antidepressant treatment with escitalopram significantly altered the expression levels of 30 miRNAs in the blood of depressed patients [16]. Furthermore, chronic administration of fluoxetine increased the level of miR-16 in the serotonergic raphe nuclei in mice. This observation was associated with decreased levels of the serotonin transporter (SERT) in monoaminergic neurons and elevated levels of synaptic serotonin (5-HT) concentration [17]. This work shows that some of the brain miRNAs may mediate the therapeutic effects of antidepressant drug treatment.

Although recent advances have enhanced our understanding of the contribution of miRNAs in pathomechanisms of depression, little is known about their role in the regulation of specific downstream molecular targets and the development of a resilient phenotype. To further explore this issue, we examined the expression levels of a set of five different miRNAs (miR-18a-5p, miR-34a-5p, miR-135a-5p, miR-320-3p, and miR-674-5p) in the hippocampus (HIP) and nucleus accumbens septi (NAcc) of stress-susceptible and resilient animals. Both brain structures are important in the processing of emotions, and thus are of particular interest in studies on stress-related mood disorders [18, 19]. All miRNAs included in this work have been reported to be significantly involved in the regulation of central nervous system homeostasis and in the development of depressive disorders. For instance, increased expression of miR-18a-5p has been observed in the brain of the stress-vulnerable F344 rat strain [20] and in the basolateral amygdala of stressed adolescent rats [21]. Moreover, we previously observed reciprocal alterations in the expression levels of miR-18a-5p in the mesocortical circuit of resilient rats [22]. Using an animal model of depression, it has been shown that the antidepressant effect of chronic duloxetine treatment was associated with increased expression levels of miR-18a-5p in the hippocampus [23]. miR-34a may modulate individual stress-coping strategies by acting on corticotrophin-releasing factors in the dorsal raphe nuclei [24] and amygdala [25]. It has also been postulated that the serum level of miR-34a may correlate with the occurrence of depression [26]. Recent studies have shown that depressed human patients had decreased expression levels of miR-135a in the blood and brain [27] and that acute stress altered the expression level of miR-135a in the amygdala of stressed mice [28]. Another study reported that dysregulation in the blood levels of the miR-320 family may accompany depression [29]. Moreover, miR-320 is a potential regulator of neuronal regeneration and neurite outgrowth in the central nervous system [30]. Finally, a preclinical study has shown that traumatic stress can cause dysregulation in the expression of miR-674 in the amygdala as well as in the serum of stressed animals [31].

Serotonin5-HT_1A_ receptors (5-HT_1A_Rs) have been proposed as key mediators of serotonergic signaling in the hippocampus [32]. 5-HT_1A_R belongs to a class of inhibitory G protein-coupled receptors and is widely expressed in the HIP, where it acts as an auto- or heteroreceptor and is located pre- and post-synaptically, respectively. Therefore, 5-HT_1A_Rs may act in a dualistic manner by controlling serotonin tone and release as well as by modulating the cellular response to this monoamine in brain regions receiving serotonergic innervations. Chronic stress reduced the levels of 5-HT_1A_Rs in different brain areas (including the HIP) in depressed patients [32] and in an animal model of depression [33]. It has been postulated that hippocampal 5-HT_1A_Rs may be a molecular factor implicated in resilience to stress and response to antidepressant treatment [34, 35]. For instance, it has been observed that Flinders stress-resistant rats had a higher density of 5-HT_1A_Rs in the CA1 region of the hippocampus compared to stress-susceptible rats [36]. Another study has shown that resilience to stress and a good prognosis of an antidepressant response are associated with lower levels of 5-HT_1A_ autoreceptors [37] but not heteroreceptors.

Dysregulated function of both brain 5-HT_1A_Rs and miRNA transcripts are important in the pathogenesis of depression. Nevertheless, knowledge about their mutual regulatory interaction in the formation of stress-resilient and stress-susceptible phenotypes is vague. In this work, we attempted to delineate differences at the level of brain miRNA that might characterize anhedonic and resilient phenotypes. Moreover, we sought to determine whether alterations at the miRNA level may influence serotonergic homeostasis in the brains of animals expressing different behavioral responses to stress. Delineation of molecular factors associated with a resilient phenotype may not only result in a better understanding of the pathogenesis of stress-related mood disorders but may also open the way to the development of new antidepressant therapeutic strategies involving pharmacological manipulations at the level of microRNA.

## Experimental Procedures

### Chronic Mild Stress Procedure

Male Wistar Han rats (Charles River, Germany) were singly housed in plastic cages (40 × 25 × 15 cm) in standard housing conditions, i.e., 22 ± 2 °C, 40% humidity, and a 12-h light-dark cycle, with water and food available ad libitum. At the age of 4 months, rats were adapted to the experimental conditions and trained to a drink palatable 1% sucrose solution for 6 weeks. After the training period, the animals were randomly divided into the control (*n* = 110) and stressed (*n* = 110) groups. Controls were housed undisturbed in a separate room. Stressed animals were subjected to two consecutive weeks of a mild stress regimen that consisted of two periods of food deprivation: 45° cage tilt, stroboscopic illumination (150 flashes per min), and intermittent illumination (lights on and off every 2 min); one period of soiled bedding, water deprivation, and paired housing. All mild stressors had a duration of 10–12 h. Sucrose consumption tests were performed at the end of each weekly stress regimen followed by 14 h of water deprivation. Sucrose intake was measured as a difference in bottle weight. Discrimination between anhedonic (*n* = 78) and resilient (*n* = 32) groups of animals was based on the retrospective analysis of changes in their sucrose solution intake under stressful conditions and related to the median split of the baseline sucrose intake of these rats. Moreover, anhedonic animals, in response to stressful conditions, decreased their sucrose intake by more than 20%, while resilient subjects did not decrease their sucrose consumption levels compared to basal sucrose intake and non-stressed littermates. For further molecular analyses, we randomly chose 16 animals per group. The remaining rats were used for another experiment and were not included in this study. All behavioral experiments were approved by the II Local Bioethics Commission (Krakow, Poland) and were carried out in accordance with EU Directive 2010/63/EU for animal experiments.

### Serum and Brain Tissue Sample Collection

Before the start of CMS, 0.5 ml of tail-vein blood was collected from each rat. Then, 24 h after the last stressor and sucrose consumption test, all animals were sacrificed by decapitation. Trunk blood samples were collected, and brains were rapidly removed from the skull. Blood samples were kept at room temperature (RT) for 30 min to clot and then centrifuged at 4 °C at × 1500*g* for 10 min. Non-hemolyzed serum samples were frozen at − 80 °C and stored until use. For autoradiographic and in situ analyses, whole brains were frozen in a mixture of dry ice and heptane and then cut into 12-μm-thick coronal sections through the dorsal (bregma, − 3.14 to − 3.80 mm) and ventral hippocampus (bregma, − 4.80 to − 5.60 mm) using a Jung CM 300 cryostat microtome (Leica, Germany) and mounted on gelatin-coated slides and air-dried. For miRNA screening and RT-PCR, Western blot, and HPLC analyses, the HIP and NAcc were dissected from rat brains, HIP was divided into dorsal and ventral parts and all brain regions were immediately frozen on dry ice in Eppendorf tubes and stored at − 80 °C until use.

### Serum Corticosterone ELISA

Serum corticosterone (CORT) levels were measured using a commercially available rat corticosterone ELISA kit (Demeditec Diagnostics, Germany) strictly according to the manufacturer’s protocol. The absorbance was determined at 450 nm. Serum CORT concentrations were calculated from a calibration curve ranging from 15 to 2250 ng/ml. To eliminate the effect of a circadian rhythm on rat serum corticosterone concentrations, tail-vein and trunk blood samples were collected from all animals between 10 a.m. and 1 p.m.

### Total RNA Including miRNA Purification

Total RNA, including miRNA from tissue and cell samples, was extracted using the miRNeasy Micro Kit. The miRNeasy Serum/Plasma kit was used for the isolation of total RNA, including miRNA, from serum samples. All purification methods were performed automatically on a QIAcube robotic workstation according to the manufacturer’s protocols (Qiagen, Germany). The purity and concentration of RNA samples were measured with a NanoDrop spectrophotometer (ThermoFisher, USA).

### Screening of miRNA Expression Changes in the HIP, NAcc, and Serum of Rats After 2 Weeks of CMS Using Custom-Made TaqMan Low-Density Cards (TLDA)

Samples of pure total RNA, including miRNA from serum (3 μl) and brain tissue (1 μg), were reverse transcribed into cDNA using the TaqMan MicroRNA Reverse Transcription Kit and custom-made TaqMan MicroRNA Assay mixture (LifeTechnologies, USA) according to the procedure for multiplexing the RT step without preamplification while using TaqMan MicroRNA Assays. Next, 6 μl of cDNA was mixed with 44 μl of pure water and 50 μl of TaqMan Universal PCR MasterMix, no AmpErase UNG, and the whole mixture was loaded into the custom-made TaqMan low-density cards (TLDA) (Life Technologies, CA, USA). Real-time PCR was performed on the QuantStudio™ 12 K Flex system (Life Technologies, CA, USA) with default cycling conditions and automatic threshold values according to the manufacturer’s protocol. Gene expression values were calculated using the delta-delta *C*_*t*_ method and were normalized to U6 transcript for brain samples and geometric means of miR-106b and let-7 g for serum samples. Supplementary material 1 contains a list of all TaqMan MicroRNA Assays used in the experiment.

### Comparative Bioinformatic Analysis

Bioinformatic analysis of the interaction between miR-18a-5p and rat 5-HT_1A_R mRNA was performed with the freely accessible online database miRWalk 2.0 [38]), which documented miR-18a-5p-binding sites within the coding sequence of 5-HT_1A_R mRNA and combined this information with two other miRNA-target prediction programs, i.e., TargetScan and miRanda. All prediction algorithms showed the miR-18a-5–HT_1A_R interaction. Gene ontology and KEGG pathway analyses of putative gene targets expressed in the hippocampal tissue and being regulated by miR-18a-5p were performed using GeneCodis3 software [39] based on calculations of a hypergeometric distribution followed by *p* value corrections for multiple testing. A list of tissue-specific genes expressed in native, non-diseased hippocampal tissue was obtained from GTEx Portal (https://gtexportal.org) on 12/2018. Expression of a particular gene in the hippocampus was considered as detectable when TPM value in the database was more than 5.

### Neuronal Cell Culture from Adult Rat Hippocampus and Immunocytochemistry

Adult hippocampal neuron cell culture was performed according to a previously published protocol [40] with minor modifications. In brief, dissected hippocampi were cut in 0.5 mm thick slices and incubated at 30 °C for 30 min in HibernateA medium without CaCl_2_ (BrainBits, USA) containing 2 mg/ml of papain (Sigma-Aldrich, Germany). Digested slices were triturated and fractionated on an Optiprep 1.320 gradient (Sigma-Aldrich, Germany). The neuronal fraction was collected, rinsed with HibernateA/B27 and centrifuged for 1 min at × 200*g*. The cell pellet was resuspended in NeurobasalA/B27 medium containing 1 μg/ml gentamycin, 0.5 mM Glutamax, and 10 ng/ml fibroblast growth factor (ThermoFisher, USA). Neurons were seeded (250,000 cells/slide) on poly-D-lysine-coated glass coverslips (Merck, Germany) and maintained for 10 days at 37 °C under a 5% CO_2_ humid atmosphere. For immunocytochemistry, cell cultures were rinsed with PBS, fixed in 4% paraformaldehyde for 30 min at 37°C and pre-blocked for 1 h at RT in PBS containing 5% bovine serum albumin and 0.1% Tween 20 (Sigma-Aldrich, Germany) followed by overnight incubation at 4 °C with the same blocking solution enriched with anti-MAP2 (neuronal marker; M9942) and anti-GFAP (astroglial marker; EP6724) primary antibodies at a final dilution of 1:500. The next day, the cells were rinsed 3 × 5 min with PBS and incubated for 1 h at RT with donkey-Cy3 (AP182C) and goat-AlexaFluor 488 (ab150117) secondary antibodies diluted 1:500 in blocking solution. Then, the cells were washed 3× for 5 min in PBS and mounted in SlowFade™ Gold Antifade Mountant with DAPI (ThermoFisher, USA). Fluorescently labeled cells were examined using an AxioImager 2 fluorescence microscope equipped with Zen 2 Pro software (Carl Zeiss MicroImaging GmbH, Germany).

### Transfection of Hippocampal Neurons with miR-18a-5p Mimic

Neuronal cells were transfected with either 20 nM miR-18a-5p mimic or Silencer® Select negative control (ThermoFisher, USA) using the N-TER™ Nanoparticle siRNA Transfection System (Sigma-Aldrich, Germany) according to the manufacturer’s protocol. Forty-eight hours post-transfection, neuronal cells were washed in ice-cold PBS and used for further molecular experiments. Silencer® Select negative control had no significant sequence similarity to rat genes and was used to verify the specificity of the miR-18a-5-HT_1A_R mRNA interaction.

### RT-qPCR Analysis

A total of 700 ng of total RNA from brain and cell samples was reverse transcribed into cDNA using a High-Capacity cDNA Reverse Transcription Kit (ThermoFisher, USA). Next, 0.5 μl of cDNA was mixed with 8.5 μl of water, 10 μl of TaqMan™ Gene Expression Master Mix and 1 μl of gene-specific TaqMan™ Gene Expression Assay (ThermoFisher, USA). The RT-PCR reaction was run on a CFX96 Touch™ Real-Time PCR Detection System (Bio-Rad, Germany) using default thermal cycling conditions. Raw *C*_*t*_ values were analyzed and normalized to *β*-actin control using the delta-delta *C*_*t*_ method. Supplementary material 1 contains a list of all TaqMan Gene Expression Assays used in the experiment.

### 5-HT_1A_R mRNA Expression: In situ Hybridization

Brain sections were fixed in ice-cold 4% formaldehyde for 10 min, washed in phosphate-buffered saline (PBS), incubated for 10 min in an ice-cold 0.1 M triethanolamine—0.25% acetic anhydride mixture and dehydrated in a graded series of alcohol followed by two 10-min incubations in chloroform. A mixture of three oligonucleotides complementary to rat 5-HT_1A_R mRNA was used as follows:

- 5′ATGAGCAACAGCGGGATATAGAAAGCGCCGAAAGTGGA3′
- 5′TGGTAGCTGAAGGTCACGTCGGAGATGCTAGTAACGTTGCCGCC3′
- 5′TGGAGTAGCCTAGCCAGTTAATTATGGCACCCAACAACGCAGG3′

Oligonucleotides were designed using BLAST software and are specifically complementary to three different 5-HT_1A_R mRNA sites to increase the detection sensitivity of the in situ technique. Terminal transferase (Fermentas, Lithuania) was used to radiolabel oligoprobes at the 3′ ends with [^35^S]dADP (Hartmann Analytic, Germany). Radiolabeled oligonucleotides were suspended at a concentration of 1 × 10^6^ dpm per 50 μl of hybridization buffer. Brain slices with applied hybridization buffer containing radiolabeled oligonucleotides were incubated for 18 h at 37 °C. After hybridization, tissue slices were washed four times in 1× SSC solution for 15 min each at 42 °C, briefly immersed in distilled water and absolute ethanol, air-dried, placed into X-ray cassettes and exposed to film plates (Kodak, Japan) for 30 days at − 20 °C. The developed radiograms were quantified using ImageGauge software (Fujifilm, Japan).

### Western Blot Analysis

Hippocampi were homogenized in buffer containing 7 M urea, 2 M thiourea, 40 mM Tris, 4% CHAPS, 65 mM DTT, and protease inhibitor cocktail (Merck, Germany) and then centrifuged at 10000*g* for 10 min at 4 °C. Supernatants were collected. Total protein concentrations were measured using Bradford reagent (Sigma-Aldrich, Germany). Twenty-five micrograms of total protein per sample was mixed with 2.5 μl of Bolt®LDS Sample Buffer denatured for 10 min at 70 °C and loaded into precast Bolt™ 4–12% Bis-Tris Plus Gel (ThermoFisher, USA). Electrophoresis was run in Bolt® MES buffer (ThermoFisher, USA) for 45 min under a constant voltage of 165 V. Separated proteins were transferred to a cellulose membrane using an iBlot™ Western Blotting System (ThermoFisher, USA) according to the manufacturer’s protocol. Protein bands of interest were visualized using an iBind™ Western Kit (ThermoFisher, USA) according to the manufacturer’s protocol and using antibody dilutions as follows: anti-5-HT_1A_R (sc-1459), 1:200; anti-*β*-actin (A5441), 1:1000; anti-glucocorticoid receptor (3660s), 1:200; anti-mineralocorticoid receptor (ab64457), 1:200 primary antibodies and IgG-HRP (sc-2350), 1:100; (A9044), 1:4000; and (ab6721), 1:1000 secondary antibodies. The signals were developed using Clarity™ Western ECL Blotting Substrate (Bio-Rad, USA) and visualized with the use of Luminescent Image Analyzer Fuji-Las 4000 (Fuji, Japan). Immunoreactive bands were quantified using ImageJ software.

### [^3^H]8-OH-DPAT Binding to 5-HT_1A_R

Brain slices were hydrated in 50 mM Tris-HCl buffer for 30 min at RT and then incubated for 1 h at RT in 50 mM Tris-HCl containing 4 mM CaCl_2_, 0.1% ascorbic acid and 2 nM [^3^H]8-OH-DPAT (Perkin Elmer, USA). To determine nonspecific radioligand binding, parallel brain slices were incubated for 1 h at RT in the same buffer enriched with 10 μM serotonin (Sigma-Aldrich, Germany). The [^3^H]8-OH-DPAT concentration corresponded to the *K*_*d*_ value [41]. Radioligand incubation was terminated by three washes in ice-cold 50 mM Tris-HCl buffer for 5 min. Tissue slices were then briefly immersed in distilled water, air-dried, and exposed to Fuji Imaging Plates (Fujifilm, Japan) with autoradiographic microscales (GE Healthcare, Germany) for 7 days. Developed autoradiograms were quantified using ImageGauge software (Fujifilm, Japan).

### High-Performance Liquid Chromatography (HPLC) of 5-HT and 5-Hydroxyindolacetic Acid (5-HIAA)

Levels of 5-HT and its metabolite 5-HIAA were measured according to a previously described method [42]. In brief, weighed hippocampi were homogenized in ice-cold 0.1 M trichloroacetic acid with 0.05 mM ascorbic acid and centrifuged at 10000 *g* for 5 min. Supernatants were filtered through RC58 cellulose membranes (Bioanalytical Systems, USA). 5-HT and 5-HIAA levels were measured on a Hypersil BDS-C18 (4 × 100 mm, 3 μm) column with the use of an HP 1050 chromatograph (Hewlett-Packard, USA). For the mobile phase, 0.05 M citrate-phosphate buffer (pH 3.5) enriched with 1 mM sodium octyl sulfonate, 3.5% ethanol, and 0.1 mM EDTA was used at a flow rate of 1 ml/min. Peak areas for 5-HT and 5-HIAA were quantified with the use of ChemStation software and were compared to the standards.

### Statistical Analysis

The results from the CMS procedure and body weight gain were analyzed with the use of repeated measures ANOVA followed by Bonferroni’s post hoc test for multiple comparisons. All biochemical and molecular results were analyzed with the use of one-way ANOVA followed by Tukey’s post hoc test. Statistical analysis of the data was performed using GraphPad Prism7 software (GraphPad Software Inc., USA). GO biological processes and KEGG pathways significantly enriched with targeted genes were calculated by hypergeometric distribution and *p* values were corrected for multiple testing.

## Results

### The Effect of 2 Weeks of CMS on Sucrose Consumption, Serum CORT Concentration, and CORT Receptor Levels in Rat HIP

**Fig. 1:**
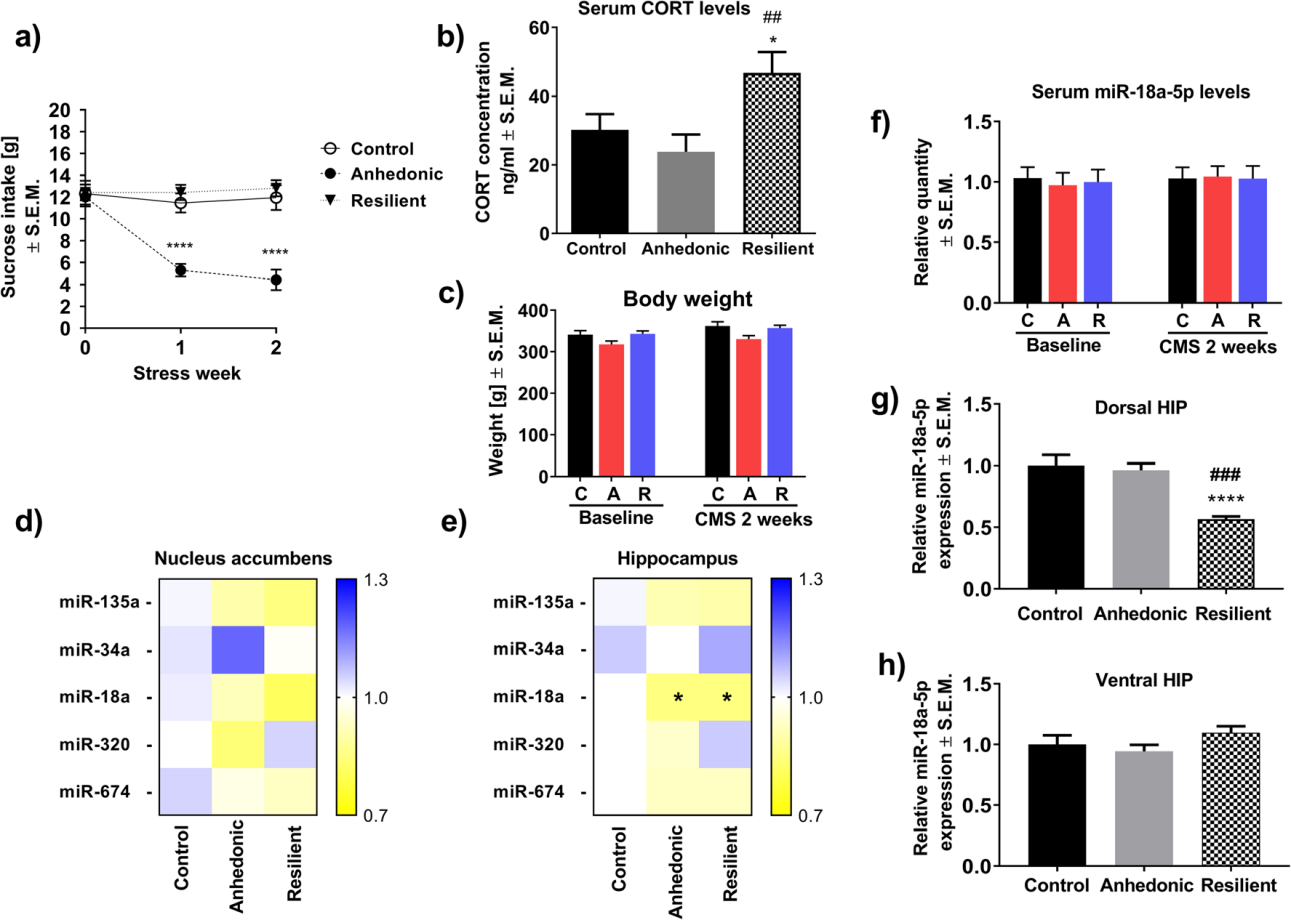
**a** The effect of 2 weeks of the CMS procedure on sucrose intake in control, anhedonic and resilient animals. Point “0” refers to sucrose intake by all examined animals before the start of CMS. Anhedonic animals significantly decreased their sucrose intake compared to control and resilient rats (*n* = 16/group), ****p < 0.0001 vs. control and resilient. **b** Elevated serum levels of CORT in resilient animals (*n* = 15–16/group): *p < 0.05 vs. control, ##*p* < 0.01 vs. anhedonic group observed after 2 weeks of CMS. **c** Time-dependent analysis of body weight gain/loss among stressed and non-stressed animals. Two-way ANOVA followed by Bonferroni’s post hoc test showed no changes in body weight in all groups of animals before and after 2 weeks of CMS (*n* = 16/group). **d** Results of the screening analysis of the expression levels of five different miRNAs in the NAcc of stressed and non-stressed animals (*n* = 10/group). **e** Results from the screening analysis of the expression levels of five different miRNAs in the HIP of stressed and non-stressed animals. One-way ANOVA followed by post hoc tests showed a significant decrease in the expression level of miR-18a in resilient and anhedonic rats compared to control littermates (*n* = 10/group), *p < 0.05. **f** Time-dependent analysis of serum miR-18a-5p levels among stressed and non-stressed animals. Repeated measures ANOVA followed by Bonferroni’s post hoc tests showed no changes in serum levels of miR-18a-5p either before the start of the CMS procedure or during stress exposure in all groups of animals (*n* = 5–9/group). **g** RT-PCR analysis of the expression level of miR18a-5p in dHIP of animals after 2 weeks of CMS. Resilient animals had significantly decreased miR-18a-5p expression in dHIP as compared to control (****p < 0.001) and anhedonic (###*p* < 0.01) littermates (*n* = 12/group). **h** RT-PCR analysis of the expression level of miR18a-5p in vHIP revealed no alterations across all animals being tested (*n* = 12/group)

Before the start of the CMS procedure, all tested animals showed the same levels of consumption of a palatable 1% sucrose solution (Fig. 1a). Repeated measures ANOVA showed a significant effect of stress (*F*_2,135_ = 34,1; *p* < 0.0001;*n* = 16), time (*F*_2,135_ = 8579; *p* < 0.0003; *n* = 16), and an interaction of stress × time (*F*_2,135_ = 7.49; *p* < 0.001; *n* = 16) on sucrose intake by the animals. Post hoc analysis revealed that anhedonic animals significantly decreased their sucrose consumption levels after the first and second weeks of the CMS procedure compared to control and resilient littermates (Fig. 1a). Statistical analysis showed no effect of stress and time on sucrose consumption in resilient subjects (Fig. 1a). Although repeated measures ANOVA suggested a significant effect of stress (*F*_2,90_ = 6.668; *p* < 0.01) and time (*F*_2,90_ = 5.255; *p* < 0.05) on body weight gain/loss of animals in CMS model, further Bonferroni’s post hoc analysis did not show any significant difference in weights between all groups of animals neither before the CMS nor after 2 weeks of stress regimen (Fig. 1c). After 2 weeks of CMS, only resilient rats exhibited significantly increased serum CORT levels (*F*_2,43_ = 5.02; *p* < 0.01; *n* = 15–16/group) compared to control and anhedonic littermates (Fig. 1b). At the same time, Western blot analysis showed normal levels of glucocorticoid (GR) and mineralocorticoid (MR) receptor proteins in the HIP of all tested animals (*n* = 4/group) (Supplementary material 2).

### The Effect of 2 Weeks of CMS on Brain and Serum miR-18a-5p Expression Levels in Rats

Screening analysis of a set of five different miRNAs (that are reported to be important in the development of the stress response) showed no differences in the expression levels of all tested miRNAs in the NAcc of rats subjected to 2 weeks of CMS (Fig. 1d). However, we found a statistically significant decrease in the expression levels of hippocampal miR-18a-5p in stressed animals (*F*_2,27_ = 4.86, *p* < 0.05; *n* = 10/group) compared to non-stressed controls (Fig. 1e). Our further RT-PCR evaluation of the results obtained from screening analysis revealed that only resilient animals had significantly decreased expression level of miR-18a-5p in the dorsal part of the hippocampus (dHIP) (*F*_2,33_ = 15.60, *p* < 0.0001; *n* = 12/group) while no changes were observed in the ventral part (vHIP) (*F*_2,33_ = 1.561, *p* > 0.05; *n* = 12/group) of above mentioned brain structure as compared to control as well as anhedonic animals (Fig. 1g–h, respectively). In order to examine whether dHIP alterations in the expression level of miR-18a-5p may have a potential to be a peripheral molecular marker of the stress resilience, we performed analysis of serum levels of miR-18a-5p among all tested animals. However, time-dependent analysis revealed no significant effect of stress (*F*_2,55_ = 1.10, *p* > 0.05; *n* = 5–9/group), time (*F*_2,55_ = 2.19, *p* > 0.05; *n* = 5–9/group), or interaction of stress × time (*F*_2,55_ = 0.82, *p* > 0.05; *n* = 5–9/group) on serum levels of miR-18a-5p in control and stressed animals before the start of the stress regimen (baseline) as well as during the 2 weeks of CMS (Fig. 1f) what indicated that changes in the expression level of miR-18a-5p in dHIP of resilient animals are brain-specific.

### Bioinformatic Analysis of Genes Expressed in HIP and Being Regulated by miR18a-5p and Their Biological Function

**Fig. 2:**
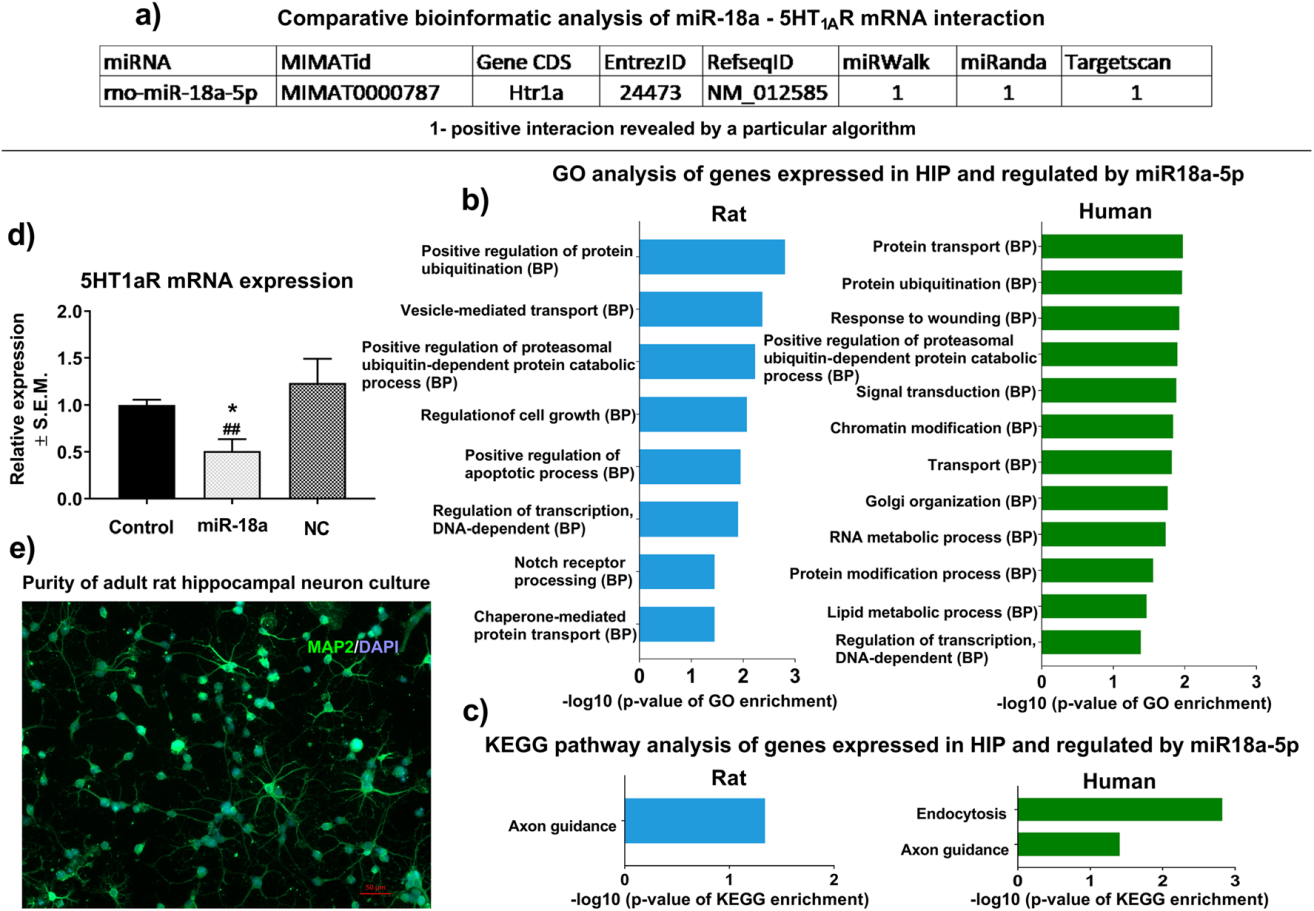
**a** Comparative bioinformatic analysis with the use of miRWalk 2.0 software shows that miR-18a-5p is a potential posttranscriptional regulator of mRNA encoding 5-HT_1A_R. 1—indicates positive predicted interaction between miR-18a-5p and mRNA encoding 5-HT_1A_R as revealed by a particular algorithm. **b** Gene enrichment analysis revealed that genes potentially regulated by miR-18a-5p and expressed in HIP are significantly involved in different biological processes associated with normal brain functioning in rats (left panel) and humans (green panel). All biological processes were calculated by hypergeometric distribution followed by *p* value correction for multiple testing. **c** KEGG pathway analysis of genes expressed in HIP and regulated by miR-18a-5p revealed that this miR is involved in the regulation of axonogenesis in both humans (right panel) and rats (left panel) and endocytosis in humans (right panel). KEGG pathways were by hypergeometric distribution followed by *p* value correction for multiple testing. **d** In vitro validation of the regulatory role of miR-18a-5p on the expression level of mRNA encoding 5-HT_1A_R. RT-PCR analysis shows that neurons transfected with 20 nM of miR-18a-5p expressed significantly lower levels of 5-HT_1A_R mRNA compared to non-transfected as well as neurons stimulated with negative control siRNA (*n* = 4–5),*p < 0.05 vs. control, ##*p* < 0.01 vs. negative control. **e** Immunocytochemical examination of the purity of the adult hippocampal neuronal cell culture. Double-staining for MAP2 and GFAP markers revealed that a vast majority of cultured cells were MAP2-positive neurons (green cells). There were no recorded GFAP-positive (orange cells) astroglial cells in the culture

In silico analysis, using the freely available online database, MirWalk 2.0 found that miR-18a-5p may potentially interact with mRNA encoding 5-HT_1A_R (Fig. 2a). Moreover, the comparative analysis showed that this potential interaction was reported by different prediction algorithms, i.e., miRWalk, miRanda, and TargetScan (Fig. 2a). Gene enrichment analysis of all potential miR-18a-5p gene targets which are expressed in HIP showed that this microRNA can be significantly involved in the regulation of biological processes relevant to the functioning of the HIP (Fig. 2b) as well as molecular pathways, such as axonogenesis, in both human and rodents (Fig. 2c).

### In vitro Validation of the Regulatory Potential of miR-18a-5p on 5-HT_1A_R mRNA Expression

To determine whether miR-18a-5p had the potential to regulate 5-HT_1A_R mRNA expression, we used primary cultures of hippocampal neurons obtained from adult rats that have high levels of endogenous 5-HT_1A_R expression. Adult neuronal cultures have a strong advantage over commonly used embryonic cells because their physiology, pharmacological properties, and even pathophysiology are more comparable to those observed in vivo in brain tissue [40]. Immunocytochemical examination of the purity of neuron cell culture showed that approximately 90% of the cells were MAP2-positive, and there were no observed GFAP-positive astroglial cells (Fig. 2e), which may overgrow the neuronal culture and therefore disturb the molecular analyses. RT-PCR analysis showed significantly decreased expression levels of mRNA encoding 5-HT_1A_R in hippocampal neurons stimulated for 48 h with 20 nM of miR-18a mimic (*F*_2,10_ = 5.41; *p* < 0.05; *n* = 4–5/group). Post hoc analysis showed that this effect was statistically significant compared to unstimulated neurons as well as to neurons transfected with 20 nM of Silencer® Select negative control (Fig. 2d).

### The Effect of 2 Weeks of CMS on the Expression Level of Hippocampal 5-HT_1A_R

**Fig. 3:**
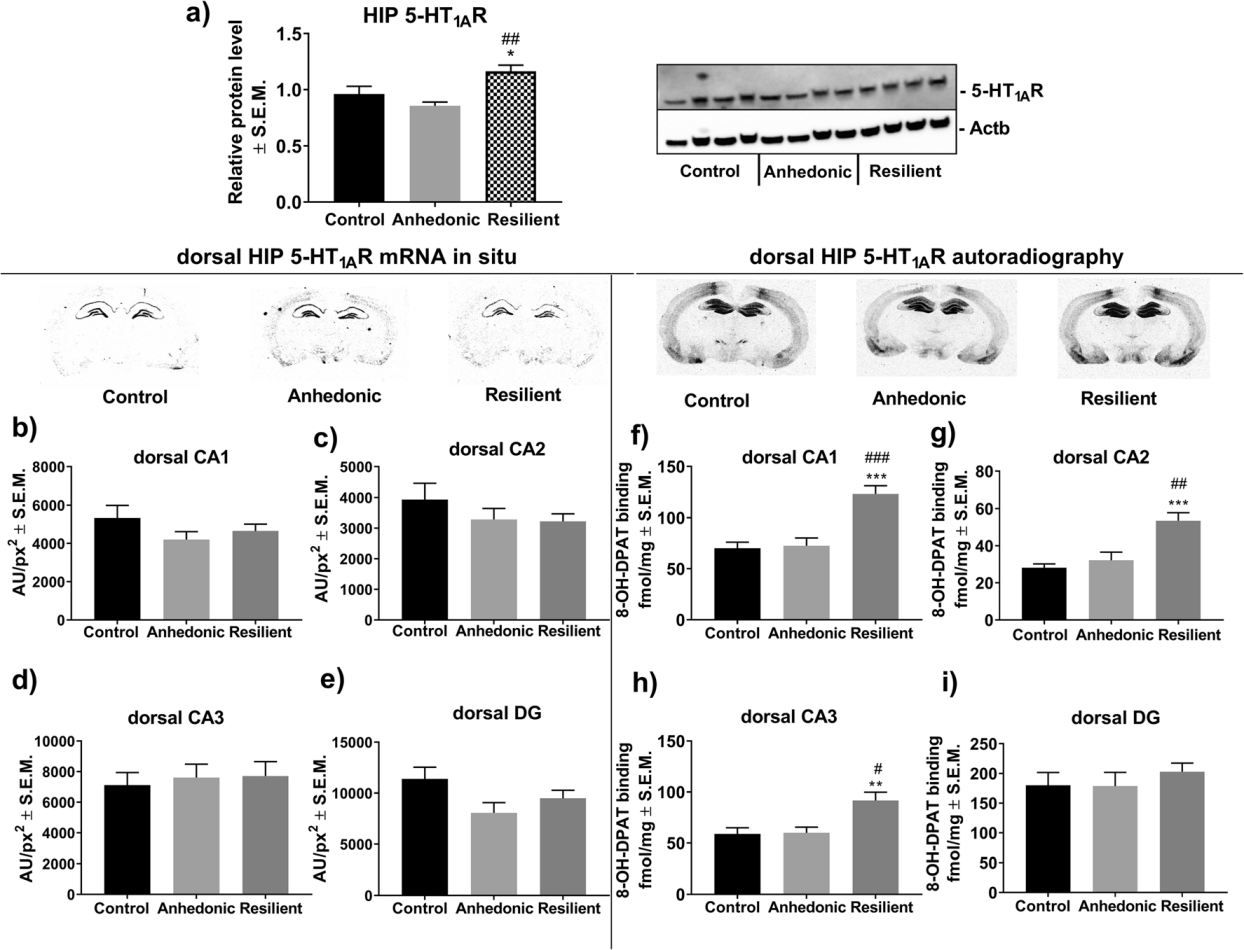
**a** Western blot analysis showed increased hippocampal 5-HT_1A_R protein levels in resilient animals; *p < 0.05 vs. control and ##*p* < 0.01 vs. resilient group of rats (*n* = 8/group). The left lower panel represents results from an in situ hybridization study of the expression level of mRNA encoding 5-HT_1A_R in **b** CA1, **c** CA2, **d** CA3, and **e** dentate gyrus (DG) subregions of the dHIP (*n* = 5–10/group). The right lower panel represents the results from [^3^H]8-OH-DPAT agonist binding to 5-HT_1A_R in **f** CA1, **g** CA2, **h** CA3, and **i** DG subregions of the hippocampus. Resilient animals exhibited increased [^3^H]8-OH-DPAT binding to 5-HT_1A_R in **f** CA1, **g** CA2, and **h** CA3 subregions of the dHIP compared to non-stressed and anhedonic littermates (*n* = 6–7/group),**p < 0.01, ***p < 0.001 vs. control; #*p* < 0.05, ##*p* < 0.01, ###*p* < 0.001 vs. anhedonic

**Fig. 4:**
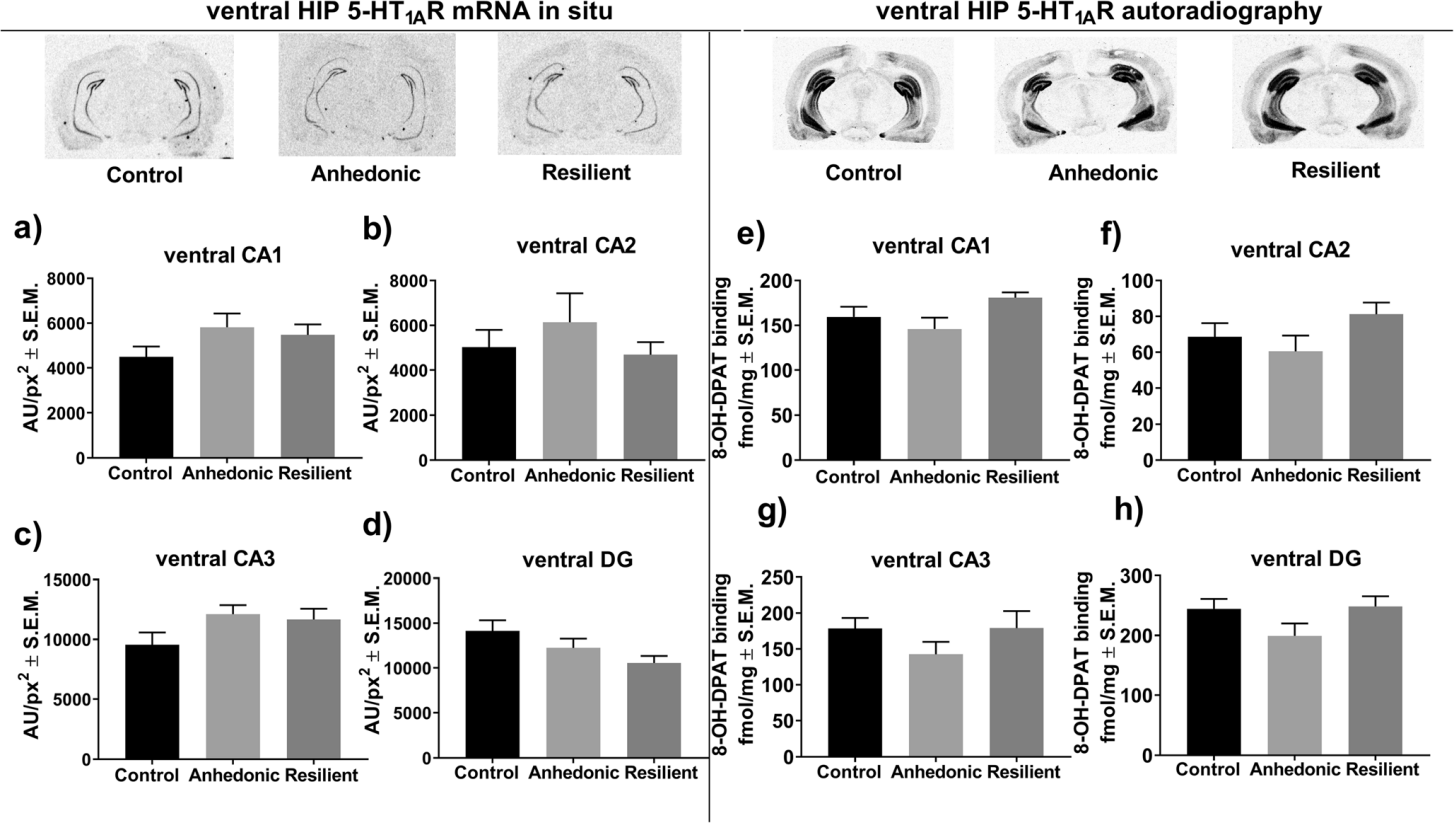
Left panel represents results from an in situ hybridization study of the expression level of mRNA encoding 5-HT_1A_R in **a** CA1, **b** CA2, **c** CA3, and **d** dentate gyrus (DG) subregions of the vHIP (*n* = 5–8/group). The right panel represents the results from [^3^H]8-OH-DPAT agonist binding to 5-HT_1A_R in **e** CA1, **f** CA2, **g** CA3, and **h** DG subregions of the vHIP (*n* = 6–9/group). One-way ANOVA showed no statistically significant alterations in mRNA expression and 5-HT_1A_R levels in all examined subregions of the vHIP in animals after 2 weeks of CMS

One-way ANOVA analysis showed a significant effect of stress exposure on overall hippocampal 5-HT_1A_R protein (*F*_2,21_ = 8.06; *p* < 0.01; *n* = 8/group) as measured by Western blot. Post hoc analysis revealed that stress-resilient subjects had significantly higher hippocampal levels of 5-HT_1A_R protein compared to those in control and anhedonic animals (Fig. 3a). Further histological examination with the use of radiolabeled 5-HT_1A_R agonist [^3^H]8-OH-DPAT showed that increased 5-HT_1A_R levels in the resilient animals were restricted to CA1 (*F*_2,17_ = 17.82, *p* < 0.001; *n* = 6–7/group), CA2 (*F*_2,15_ = 12.90, *p* < 0.001; *n* = 6/group) and CA3 (*F*_2,17_ = 7.79, *p* < 0.01; *n* = 6–7/group) subregions of the dHIP compared to those in control and anhedonic rats (Fig. 3f–h, respectively). On the other hand, the autoradiographic study did not show altered 5-HT_1A_R levels in all examined subregions of the vHIP (Fig. 4e–h). Furthermore, we observed no changes in the expression levels of mRNA encoding 5-HT_1A_R in all examined anatomical areas of the dHIP (Fig. 3b–e) and the vHIP (Fig. 4a–d) in either the anhedonic or resilient groups of rats compared to the control group.

### The Effect of 2 Weeks of the CMS Procedure on Hippocampal 5-HT and 5-HIAA Levels in Animals

**Fig. 5:**
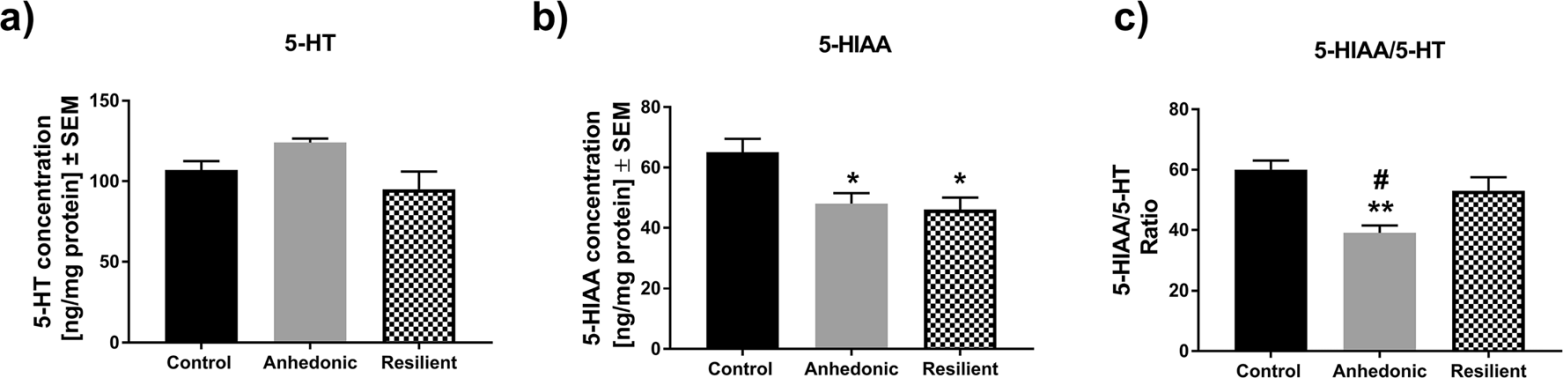
The effect of 2 weeks of CMS on serotonergic balance in the HIP of stressed and non-stressed rats. **a** Represents hippocampal 5-HT concentrations in all groups of animals; **b** 2 weeks of CMS was associated with a significant decrease in the hippocampal 5-HIAA levels in anhedonic and resilient animals, *p < 0.05 vs. control (*n* = 4/group); **c** anhedonic animals exhibited a significantly decreased 5-HT/5-HIAA ratio in the HIP compared to control and resilient rats (*n* = 4/group), **p < 0.01 vs. control and #*p* < 0.05 vs. resilient group

HPLC analysis showed no differences in hippocampal serotonin (5-HT) concentrations (Fig. 5a) across all groups of animals. However, we observed that both anhedonic and resilient animals exhibited significantly decreased levels of intraneuronal serotonin metabolite 5-hydroxyindoleacetic acid (5-HIAA) (*F*_2,9_ = 6.74; *p* < 0.01; *n* = 4/group) compared to levels in non-stressed littermates (Fig. 5b). Moreover, only anhedonic individuals showed a diminished ratio of 5-HIAA/5-HT in the hippocampus (*F*_2,9_ = 9.66; *p* < 0.005; *n* = 4/group) compared to the control and resilient groups of rats (Fig. 5c), which suggested that anhedonic but not resilient animals expressed lowered hippocampal 5-HT turnover in response to stressful conditions.

## Discussion

In the present study, we focused on finding potential epigenetic factors and their downstream molecular targets that may be associated with the development of resilient behavior. The resilient phenotype is characterized in individuals who, despite experiencing chronic stress, maintain a positive mood and general psychological balance. Because recent behavioral experiments have demonstrated that most commonly used animal models of depression can generate behaviorally resilient animals [11, 43–45], it has become possible to expand the field of preclinical research on the molecular and biochemical mechanisms underlying “stress-resistant” behaviors. In this work, we used the CMS paradigm, a well-validated animal model of depression [46–48] that, by using different long-lasting and mild socio-environmental stressors, mimics pro-depressive conditions observed in human life. Based on the analysis of sucrose intake by the rats, which is a core marker of responsiveness to reward stimuli, we were able to discriminate resilient and anhedonic animals (Fig. 1a). Decreased sucrose intake by stress-vulnerable animals and, at the same time, a lack of differences in body weight gain between all tested groups of rats during 2 weeks of CMS (Fig. 1c) indicate that this test measures hedonistic aspects of behavior rather than overall food intake. The CMS paradigm, by generating subjects with different behavioral coping strategies, mimics the natural heterogeneity of stress responses observed among humans. Further biochemical analysis revealed that resilient, but not anhedonic, animals exhibited significantly elevated serum CORT level after 2 weeks of CMS (Fig. 1b). At the same time, all groups of rats had normal levels of GR and MR in the hippocampus (Supplementary materials 2). Fine-tuned CORT release increases the energetic and metabolic rate of the organism and promotes coping with adversity [49]. Therefore, increased serum levels of CORT in resilient rats may reflect their metabolic arousal, which in turn may contribute to a more effective coping strategy in the face of stress. These observations are consistent with our previous work where we showed that resilient animals responded to 2 weeks of CMS by an elevated level of serum CORT and this response was normalized to the control level during long-lasting stress, i.e., 7 weeks of CMS [11]. Moreover, recent reports have shown that depression, as well as vulnerability to stress (in animal models of depression), may be associated not only with exacerbated hypothalamus-adrenal-pituitary (HPA) axis activity but also with blunted cortisol/corticosterone release [50–52]. Normal levels of hippocampal GR and MR observed across all animals being tested in our study may indicate that the CORT response in the resilient animals was not exaggerated and thus beneficial. It should be mentioned that we measured GR and MR protein levels in whole hippocampi, and therefore some potential changes specific only for dHIP or vHIP could have been diluted by using pooled tissue samples. It has been reported, however, that basal level of GR is higher in dHIP while MR level is higher in vHIP [53]. Thus, by measuring the levels of both receptors in HIP samples, it can be postulated that potential changes would come from the region with higher expression of either GR or MR. Nevertheless, the effect of different stress phenotype on the expression of GR and MR in subregions of the hippocampus needs further detailed research.

miRNA expression screening analysis suggested that stressful conditions may alter the expression level of miR-18a-5p in the HIP of experimental animals (Fig. 1e). A panel of a set of five miRNAs was chosen based on a literature survey and based on our previous results showing that all of the miRNAs examined here potentially regulate serotonergic transmission in the mesocortical circuit of resilient animals [22]. Our further RT-PCR evaluation of screening analysis revealed that only resilient animals had significantly decreased the expression level of miR-18a-5p as compared to control and anhedonic animals and this alteration was exclusively restricted to the dHIP (Fig. 1g). It is worth noting that, in our previous study, we observed reduced the expression level of miR-18a-5p in the prefrontal cortex (PCx), together with increased expression of the same miR in the ventral tegmental area (VTA) of resilient animals [22]. Both brain structures have influential effects on different parts of the hippocampus. vHIP receives reach innervation from the VTA [53] while dHIP mainly from the mPCx [54]. Similar direction of changes in the expression level of miR-18a-5p observed in the dHIP and PCx may confirm functional and anatomical connectivity of both brain areas. Our findings suggest that various regulations of miR-18a-5p expression in resilient subjects may depend on a unique transcriptomic landscape of a particular brain structure or even cell type.

Different gene expression patterns, anatomical projections, as well as specific functional outcomes suggest that hippocampus can be divided into at least two separate zones, i.e., the dHIP and the vHIP. The dHIP has been classified as a region mainly responsible for processing different types of memory as well as for cognitive functions and contextual fear. On the other hand, the functioning of the vHIP has been correlated with brain structures involved in stress and emotion [55–57]. Thus, both parts of the hippocampus are profoundly but differentially involved in processing of a wide range of psycho-vegetative aspects related to coping with stressful events. In our work, we observed that CMS had a strong impact on dHIP of resilient animals. This observation is consistent with previous work which has also reported that chronic unpredictable stress had a different impact on the structure and function of the dHIP versus vHIP in rats [58]. Another recent study by Liu and co-workers has shown that chronic social defeat stress caused post-stress microstructural alterations in the right dHIP of resilient mice. This indicates that the dHIP may be important in stress-resilient phenotypes [59].

According to our bioinformatic and in vitro analyses, miR-18a-5p has the potential to post-transcriptionally and negatively regulate mRNA encoding 5-HT_1A_R (Fig. 2a, d), which is highly expressed in the HIP but not in the NAcc. These observations indicated that regulatory features of miR-18a-5p are important in the functioning of the dHIP under stress. It is also important to note that the nucleotide sequence of miR-18a-5p is conserved among mammals. This fact makes miR-18a-5p a good candidate in translational studies because we can find the same miR-18a-5p nucleotide sequence, and therefore similar biological properties among rodents and humans. This conclusion can be supported by our gene ontology analyses which showed that miR-18a-5p may regulate a set of genes involved in biological processes, such as axonogenesis, which are important in normal hippocampal functioning in both human and rodents (Fig. 2b–c). miRNAs are described as negative posttranscriptional regulators that can inhibit the translation of targeted mRNA or decrease its level [8]. However, decreased cellular levels of miRNA may not result in higher expression of targeted mRNA. This is because miRNAs do not interfere with the transcriptional activity of the cell. This phenomenon may explain our results showing that a lowered level of miR-18a-5p in the dHIP of stress-resilient rats was accompanied by an increased level of 5-HT_1A_R protein, but not mRNA (Fig. 3a–i). Interestingly, we did not observe altered expression of miR-18a-5p in vHIP which was accompanied by normal levels of mRNA (Fig. 4a–d) and 5-HT_1A_R protein (Fig. 4e–h) in the abovementioned hippocampal substructure. This observation suggests that molecular changes at the level of miR-18a-5p and its downstream target 5-HT_1A_R in the dHIP, but not vHIP, may be particularly important in the development of resilient phenotype. Our results partially supported other researches by Pan and co-workers who showed that chronically stressed animals had significantly decreased expression levels of miR-18a-5p in the hippocampus, which was reversed by chronic duloxetine treatment [23]. In their study, authors did not differentiate stressed animals into vulnerable and resilient groups, which could mask some molecular changes specific for each behavioral trait. Chronic treatment with duloxetine—a selective serotonin and noradrenalin reuptake inhibitor—may alter hippocampal 5-HT_1A_R density, what in turn may evoke a molecular response at the level of miR-18a-5p expression. However, this phenomenon needs further research, especially that pharmacological profile of duloxetine is wide, and thus may secondarily influence many different molecular pathways including miRNA transcription.

We also analyzed whether decreased expression of miR-18a-5p in the brain may be reflected in the peripheral blood of stressed animals. To test this, we collected serum samples from all tested animals before the start of the CMS and after 2 weeks of the stress procedure. However, we did not find any change in the serum level of miR-18a-5p among all animals (Fig. 1f). This observation suggests that stress specifically influences only brain-derived miR-18a-5p.

Among all currently known serotonin receptors, 5-HT_1A_Rs are most abundantly expressed in the hippocampus [60]. The important involvement of hippocampal 5-HT_1A_Rs in the pathomechanisms of stress-related mood disorders has been widely described in clinical and preclinical studies. Jovanovic and co-workers, by using fMRI analysis, have shown reduced 5-HT_1A_R binding in the anterior cingulate cortex and the hippocampus of chronically stressed persons compared to non-stressed subjects [61]. Animal studies seem to confirm the involvement of 5-HT_1A_R activity in the pathogenesis of depression. For instance, 5-HT_1A_ heteroreceptor depletion in developing mice resulted in decreased mobility and increased behavioral despair in adulthood [62]. We revealed that resilient animals had significantly increased 5-HT_1A_R in the CA1 (Fig. 3f), CA2 (Fig. 3g), and CA3 regions of the dHIP (Fig. 3h). Our observations are inconsistent with the previous study which has reported that activation of 5-HT1aRs in the dHIP by 8-OH-DPAT—a potent 5-HT1aR agonist or by treatment with imipramine attenuated the development of learned helplessness. Thus, activation of post-synaptic 5-HT1a receptors in the dHIP mediated adaptation to severe inescapable stress [63]. Other works have shown that that intra-hippocampal infusion of sub-effective doses 8-OH-DPAT enhanced the antidepressant effects of fish oil in supplemented animals [64]. Moreover, it has been reported that 5-HT_1A_ receptor density was significantly higher in the CA1 region of the hippocampus in the Flinders stress-resistant line of rats (FRL) compared to stress-sensitive animal strains [36]. Therefore, the increased level of hippocampal 5-HT_1A_R in resilient animals may represent a molecular marker that exerts a protective action on limbic functioning and serotonergic balance. To explore this phenomenon, we measured the concentration of 5-HT and its metabolite 5-HIAA in the hippocampi of animals subjected to 2 weeks of CMS. We found that both groups of stressed rats had lower levels of 5-HIAA (Fig. 5b) with no changes in 5-HT concentration (Fig. 5a). Additionally, we found that only anhedonic animals exhibited a reduced hippocampal 5-HT/5-HIAA ratio, which reflects the diminished serotonergic turnover observed in vulnerable rats (Fig. 5c). This observation is consistent with previous experiments showing decreased serotonin turnover in different brain areas of bulbectomized mice in an animal model of depression [65]. Resilient animals, in contrast, exhibited a normal 5-HT/5-HIAA ratio, and thus maintained serotonergic homeostatic balance in the face of stress. To sum up, we showed that the resilient (but not anhedonic) phenotype is associated with increased 5-HT_1A_R levels exclusively in the dHIP. This molecular characteristic may be under the regulatory control of hippocampal miR-18a-5p expression. As a result, these two molecular factors may contribute to maintaining hippocampal serotonergic balance in the face of adversity as observed in resilient animals.

## Electronic Supplementary Material

Supplementary material 1 The complete list of all TaqMan miRNA and Gene Expression Assays used in the RT-PCR experiments. (PNG 269 kb)

High resolution image (TIF 1467 kb)

Supplementary material 2 Western blot analysis of GR and MR protein levels in the hippocampi of rats subjected to two weeks of CMS (*n* = 4/group). No significant changes were observed in the levels of hippocampal GR and MR in all groups of animals. (PNG 268 kb)

High resolution image (TIF 1087 kb)
