# Supplementary figures and images for: Resilient Phenotype in Chronic Mild Stress Paradigm Is Associated with Altered Expression Levels of miR-18a-5p and Serotonin 5-HT1a Receptor in Dorsal Part of the Hippocampus
Source: Mol Neurobiol. 2019 May 16;56(11):7680–93. doi: 10.1007/s12035-019-1622-2 (PMC6815272; doi:10.1007/s12035-019-1622-2)

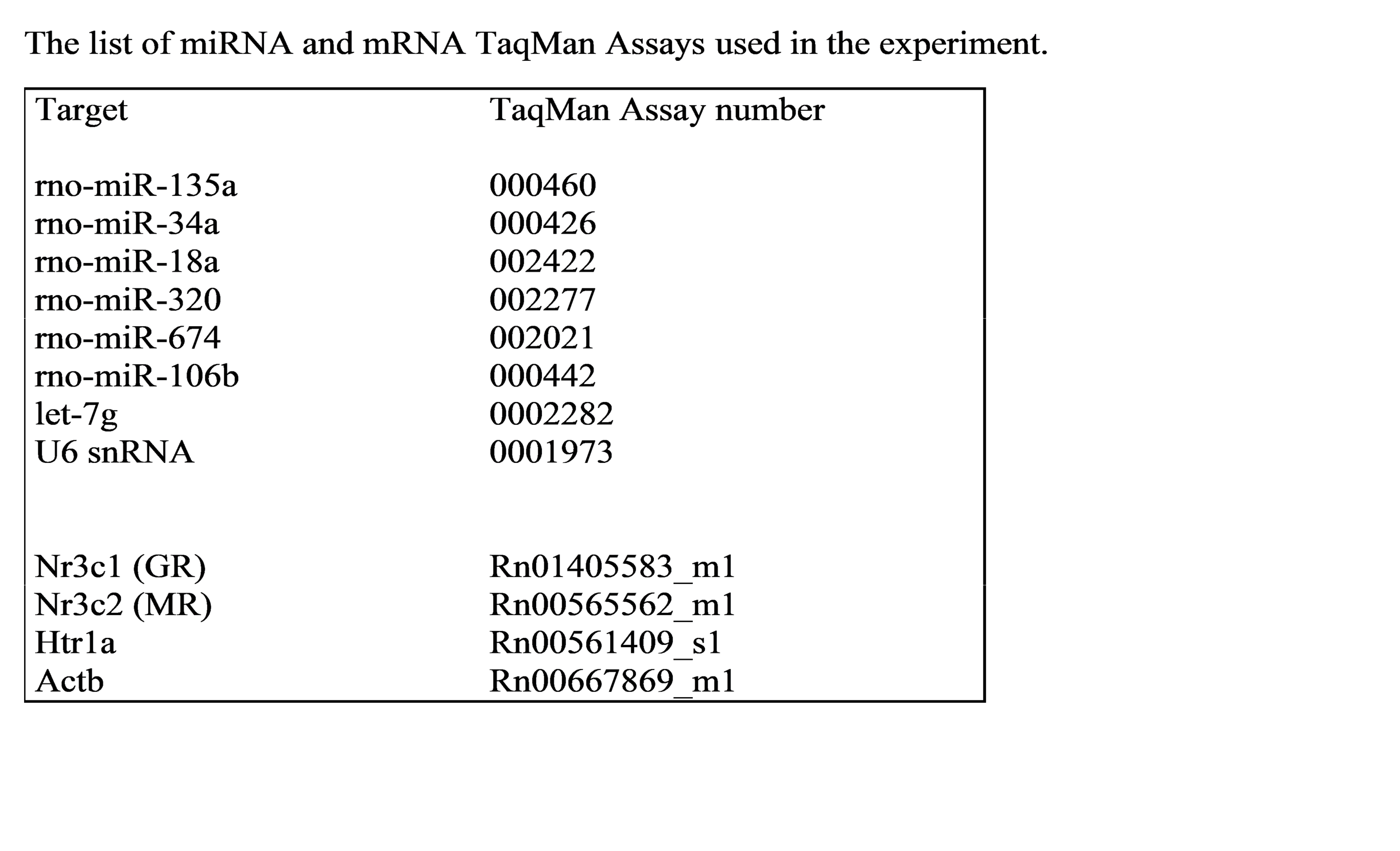

Supplement: Supplementary file 1 — The complete list of all TaqMan miRNA and Gene Expression Assays used in the RT-PCR experiments. (PNG 269 kb) [file 12035_2019_1622_Fig6_ESM.png]

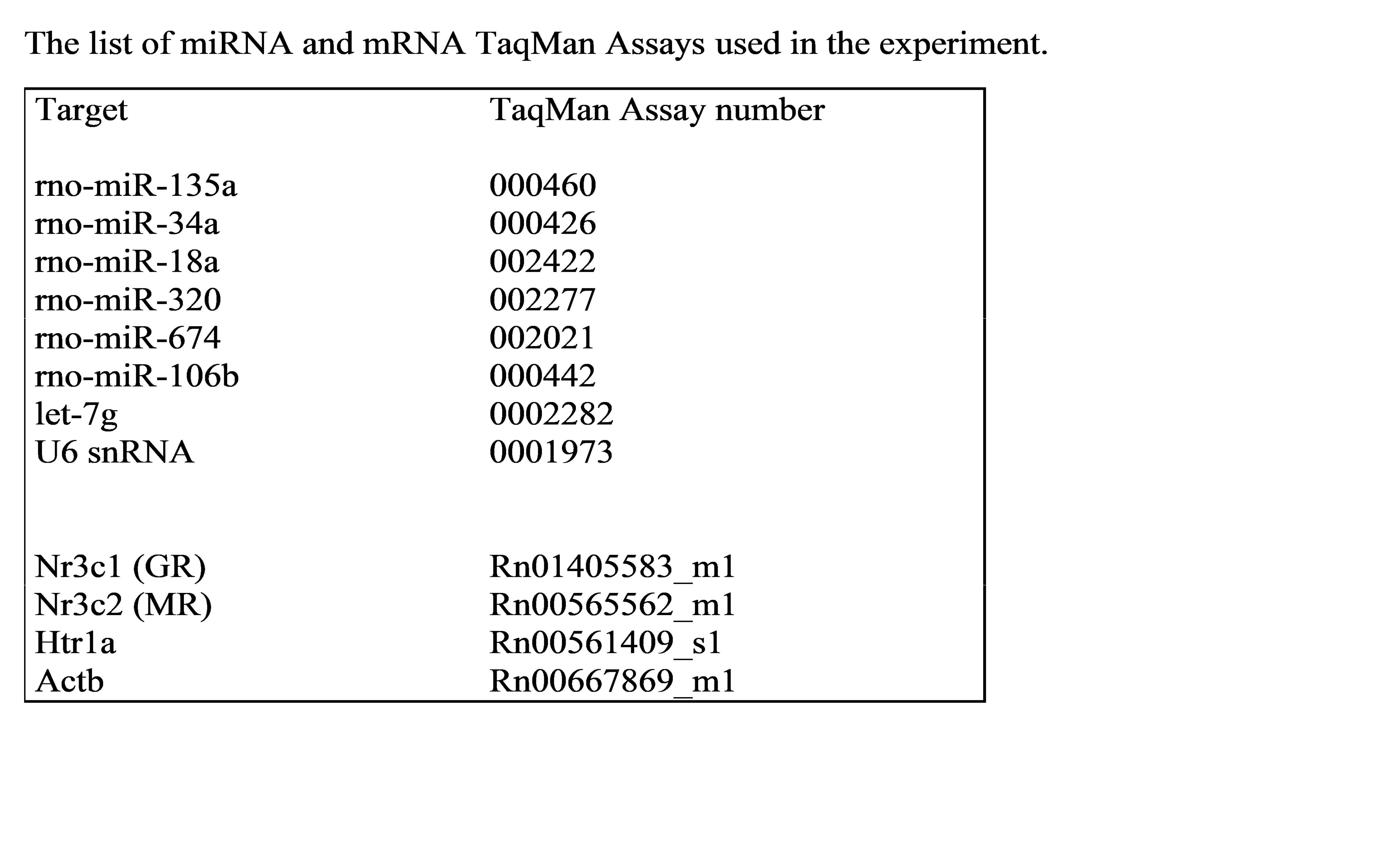

Supplement: Supplementary file 2 — High resolution image (TIF 1467 kb) [file 12035_2019_1622_MOESM1_ESM.tif]

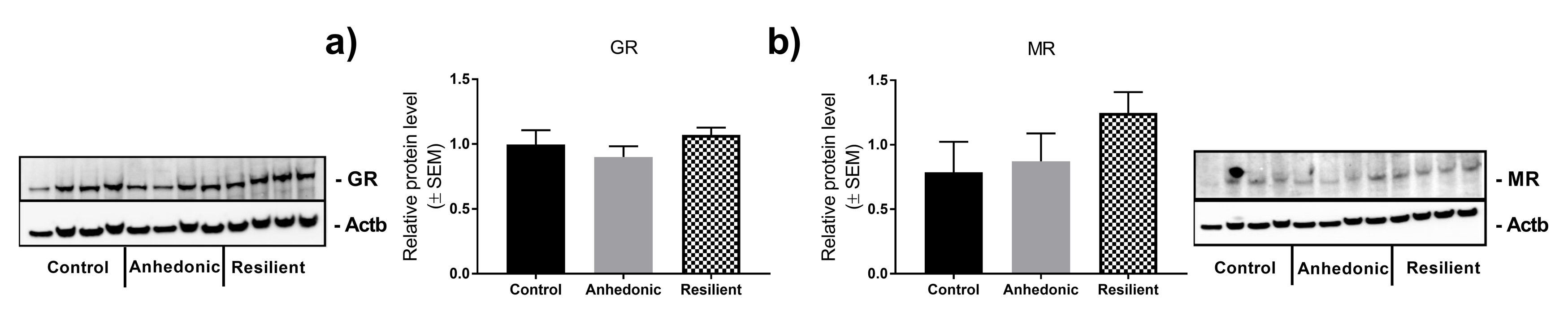

Supplement: Supplementary file 3 — Western blot analysis of GR and MR protein levels in the hippocampi of rats subjected to two weeks of CMS (n = 4/group). No significant changes were observed in the levels of hippocampal GR and MR in all groups of animals. (PNG 268 kb) [file 12035_2019_1622_Fig7_ESM.png]

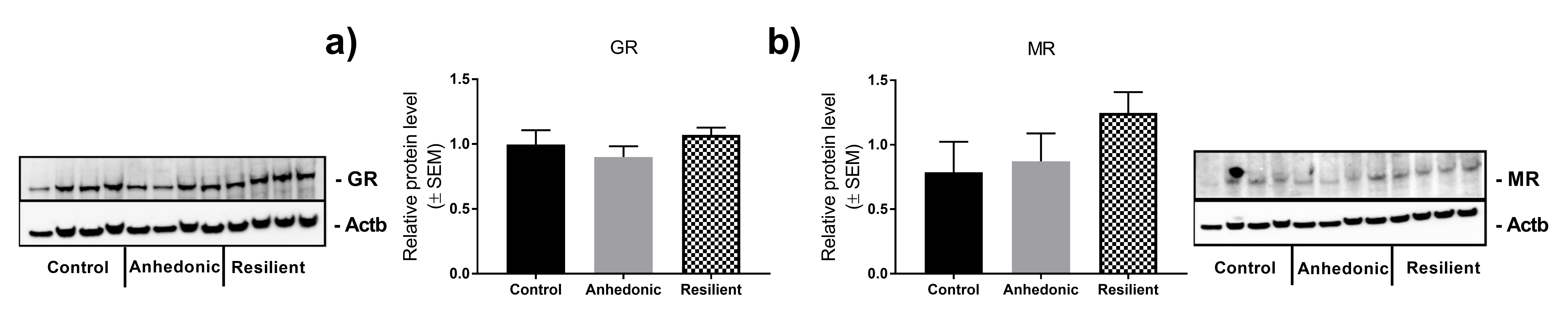

Supplement: Supplementary file 4 — High resolution image (TIF 1087 kb) [file 12035_2019_1622_MOESM2_ESM.tif]
